# Monogenic Epilepsies in Adult Epilepsy Clinics and Gene-Driven Approaches to Treatment

**DOI:** 10.1007/s11910-025-01413-x

**Published:** 2025-05-17

**Authors:** Lisa M. Clayton, Angeliki Vakrinou, Simona Balestrini, Sanjay M. Sisodiya

**Affiliations:** 1 Department of Clinical and Experimental Epilepsy, UCL Queen Square Institute of Neurology, Box 29, Queen Square, London, WC1N 3BG UK; 2 Chalfont Centre for Epilepsy, Chalfont St Peter, UK; 3 Department of Neuroscience, Pharmacology and Child Health, University of Florence, Florence, Italy; 4 Neuroscience and Medical Genetics Department, Meyer Children’s Hospital IRCCS, Florence, Italy

**Keywords:** Genetic epilepsy, Developmental and epileptic encephalopathy, Whole genome sequencing, Adults with epilepsy, Gene-driven, Precision medicine

## Abstract

**Purpose of review:**

Genetic factors play an important contribution to the aetiology of epilepsy and may have implications for management. Whilst the study of monogenic epilepsies has predominantly centred around children, there is a critical need to understand the burden of monogenic epilepsies in adults. This understanding is essential to steer the application of genetic testing and to facilitate access to gene-driven therapies in adults with epilepsy.

**Recent findings:**

The yield of diagnostic genetic testing in adults with epilepsy and neurodevelopmental disorders is similar to that in children (ranging from 23–50%). Distinct causal genes underlie the most common monogenic epilepsies identified in adulthood compared to childhood, although *SCN1A* is the most commonly implicated gene across both populations. Genetic diagnoses made in adults with epilepsy frequently have direct implications for clinical management. However, very few gene-driven therapies are supported by evidence from formal studies.

**Summary:**

Genetic testing should be considered in adults with unexplained epilepsy and may have important implications for management, including the potential for gene-driven therapies. However, further work is needed to understand the outcomes of gene-driven therapies in adults with epilepsy.

**Supplementary Information:**

The online version contains supplementary material available at 10.1007/s11910-025-01413-x.

## Introduction

A genetic contribution to the aetiology of epilepsy has long been understood, underpinned by evidence from genetic epidemiological, family, and twin studies, and far predates the current “genomics era”. However, recent progress in gene discovery has provided new perspectives and a deeper understanding of the genetic architecture of the non-acquired epilepsies [1]. In recognition of this, genetic factors are included as an important aetiological category in the most recent ILAE framework for classification of the epilepsies [2] and should be considered early in the presentation.

The genetic architecture of the epilepsies is diverse, with the contribution of various genetic aetiologies differing depending on the epilepsy type or syndrome. Genetic aetiologies of the epilepsies encompass monogenic, chromosomal, and mitochondrial disorders, as well as polygenic contributions where genetic variants (each with low to moderate effect size) across multiple genes collectively confer risk of disease, alongside epigenetic and environmental influences. Both rare and common genetic variation play a role across the epilepsies, and it is becoming increasingly apparent that variation across the genome, spanning a range of effect sizes, collectively shapes an individual's phenotype [3, 4]. In addition to germline genetic variants (i.e. those present in all cells of an affected individual), brain-restricted somatic variants, that arise post-zygotically during cortical development, have recently been shown to be an important cause of focal epilepsies, particularly in those associated with malformations of cortical development (MCD) [5].

This review will focus on the monogenic epilepsies that may be encountered in adult epilepsy clinics, with a discussion of gene-driven approaches to treatment.

## The Monogenic Epilepsies and “Epilepsy-Associated Genes”

The first epilepsy-associated gene, *CHRNA4,* was discovered in 1995 [6], and thus *CHRNA4*-related autosomal dominant sleep-related hypermotor epilepsy (in today’s language) became the first “monogenic epilepsy”. The monogenic epilepsies are those in which variation within a *single* gene is understood to have a major contribution to the overall clinical phenotype. This is in contrast to the genetic architecture of the majority of non-acquired epilepsies (including the idiopathic generalised epilepsies (IGEs) and non-acquired focal epilepsies (NAFEs)) which is understood to be complex and polygenic in most cases [7–11]. Since the discovery of *CHRNA4*, gene discovery in the epilepsies has increased substantially, with recently published lists of “epilepsy-associated genes” typically including several hundreds of genes [12–14]. Whilst there is no established definition of what constitutes an “epilepsy-associated gene” they are generally acknowledged to be genes in which variation has been associated with causing epilepsy or a syndrome in which epilepsy is a core feature [12–14]. Depending on the curation criteria and intended purpose (e.g. research or clinical use), “epilepsy-associated gene” lists vary, including in the numbers of genes included which are primarily associated with neurodevelopmental disorders, metabolic disorders, or cerebral malformations in which epilepsy is a recognised but not universal feature [12, 14]. It should be noted that the level of evidence supporting definitive gene-disease associations differs across the published “epilepsy-associated genes”. This is particularly important to consider when evaluating genetic variants for clinical purposes, as there may be limited evidence supporting disease causation for some genes. Over time, as evidence accumulates, these associations are either strengthened or refuted [15], and considerable efforts are ongoing to robustly establish definitive gene-disease relationships in the epilepsies [16, 17].

The incredible growth in the discovery of epilepsy-associated genes has largely been driven by the advent and availability of next-generation sequencing (NGS) technology, and the ability to rapidly and cost-effectively sequence the entire human genome (whole genome sequencing (WGS)), or coding portions of the genome (whole exome sequencing (WES)). An additional driver of gene discovery in the epilepsies followed discoveries that de novo variants (i.e. genetic variation that arises for the first time in an individual and is not inherited from a parent), were a major cause of the developmental and epileptic encephalopathies (DEEs) [18–20]. Currently, the majority of known epilepsy-associated genes are those that are associated with the DEEs, or early-onset neurodevelopmental and metabolic disorders of which epilepsy is a recognised feature [12, 14]. In a recently curated list of > 900 epilepsy-associated genes, 90% were considered to be associated with DEEs or neurodevelopmental/metabolic disorders where epilepsy is an associated feature. The remaining 10% of genes included those associated with genetic generalised epilepsies (GGE), NAFE, progressive myoclonic epilepsies (PME), and structural malformations (including MCD) associated with epilepsy [12].

## Monogenic Epilepsies Encountered in Adult Clinics

Much of our current understanding of the monogenic epilepsies comes from studies of children, particularly those with severe early onset epilepsies such as the DEEs. Early diagnosis and improvements in care mean that most of these children now survive into adulthood, and will therefore require care in adult epilepsy/neurology services [21]. However, these epilepsies represent only a small proportion of those encountered in adulthood, and monogenic aetiologies of other epilepsy syndromes are also important to consider.

Adolescents may transition into adult services with an established genetic diagnosis. However, many adults currently living with epilepsy will not have benefited from recent genetic diagnostic discoveries [22–24]. As gene-driven therapies in the epilepsies begin to emerge, it is becoming increasingly important that genetic aetiologies are considered in those with compatible phenotypes. For those caring for adults with epilepsy, it is essential to have an understanding of the spectrum of monogenic epilepsies that may be encountered in adulthood, including recognising which individuals may benefit from genetic testing and being aware of the limitations of *diagnostic* genetic testing in certain epilepsy syndromes.

### Genetic Generalised Epilepsies

The GGEs are a broad group of common and rare epilepsy syndromes, characterised by generalized seizure types and generalized spike-wave activity on EEG [25]. Whilst a genetic contribution to these epilepsies is clear, the genetic architecture is recognised to be complex and polygenic in most cases, with both common [26] and rare [27, 28] genetic variation contributing to disease risk.

Within this group, the IGEs are the most common, accounting for 15–20% of all epilepsies [25, 29]. Monogenic causes of IGE are rare, and are identified in only 1–2% of individuals [30]. Implicated genes include *GABRA1 *[31], *GABRG2 *[32], and *SLC2A1 *[33], all of which are also associated with a wide spectrum of other epilepsy syndromes, including DEEs [34–36]. These variants may arise de novo or be inherited, often showing incomplete penetrance and phenotypic heterogeneity within families carrying the same variant.

It is worth noting that in around 3% of individuals with IGEs, recurrent copy number variants (CNVs) (including at 15q13.3 and 16p13.11) are identified, which may arise de novo or be inherited from an affected or unaffected parent [37–39]. Whilst these CNVs increase an individual’s risk of developing an IGE, they are not understood to be wholly causal variants, but instead confer a moderate contribution to an individual’s overall polygenic risk of epilepsy [37].

### Non-Acquired Focal Epilepsies (NAFEs)

The NAFEs are focal epilepsies that are not caused by a known acquired factor (such as infection or stroke). They encompass a wide range of sporadic and familial epilepsy syndromes with onset from the neonatal period to adulthood. They may be non-lesional (i.e. without causal structural abnormalities on brain imaging) or associated with structural abnormalities such as MCD. The NAFEs are genetically heterogeneous with both common [26] and rare [40] genetic variation contributing to disease risk.

Around 30 genes have been associated with monogenic NAFEs, many of which are pleiotropic and linked with multiple epilepsy types [12]. The likelihood of identifying a monogenic cause of a NAFE varies depending on the specific epilepsy syndrome (Table 1), as well as other clinical factors, including younger age of epilepsy onset [41, 42]. Outside of the familial focal epilepsy syndromes (Table 1), monogenic causes of NAFE are infrequently found, with diagnostic yields (including in cohorts of adults with NAFE) ranging from 0 – 12% [41, 43–48] (Supplementary Table 1).

**Table 1:** Familial Focal epilepsy syndromes with known monogenic causes

Focal epilepsy syndrome	Monogenic causes^b^	Age of onset (and remission)
Self-limited (familial)^a^ neonatal epilepsy	KCNQ2 (identified in > 80%), *KCNQ3*	Onset: first days of life Remission: by 6 months
Self-limited familial neonatal-infantile epilepsy	*SCN2A* (identified in > 80%), *KCNQ2*	Onset: first days of life – 23 months Remission: by 12–24 months
Self-limited (familial)^a^ infantile epilepsy	*PRRT2* (identified in > 80%), *SCN8A*	Onset: 3 – 23 months Remission: in the first years of life
Autosomal dominant sleep-related hypermotor epilepsy (ADSHE)	*CHRNA4, CHRNA2, CHRNB2, DEPDC5, NPRL2, NPRL3, KCNT1* (Identified in ~ 19% of familial and ~ 7% of sporadic cases)	Onset: typically first two decades (but ranges from infancy to late adulthood)
Autosomal dominant epilepsy with auditory features (ADEAF)	*LGI1, RELN, DEPDC5, SCN1A* (Identified in *15–50%* of familial and *2–5%* of sporadic cases)	Onset: adolescence or early adulthood
Familial mesial temporal lobe epilepsy (FMTLE)	*DEPDC5* (Identified in < *5%* of cases)	Onset: adolescence or early adulthood
Familial focal epilepsy with variable foci (FFEVF)	*TSC1, TSC2, DEPDC5, NPRL2, NPRL3* (Unknown^c^ (*DEPDC5* variants identified in 60% in one study of 5 families))	Onset: typically first two decades (but ranges from infancy to adulthood)

Table adapted from [54]; ^a^”familial” is used when a family history of the same syndrome is present; ^b^most commonly reported genes provided, list not exhaustive; ^c^The proportion of cases where a monogenic cause is identified is unknown due to small study sizes, biases in study population selection, and challenges with obtaining large enough pedigrees to fulfil diagnostic criteria for FFEVF (i.e. demonstrating a family history of individuals with focal seizures that arise from cortical regions that differ between family members); AR = autosomal recessive.

### GATOR1-Relate Focal Epilepsies

Both germline and somatic variants in genes encoding components of the mechanistic target of rapamycin (mTOR) pathway contribute to a spectrum of epilepsies (including NAFEs) and MCD (for a review see [49]). Variants in the GTPase-activating protein activity towards Rags 1 complex (GATOR1) family of genes (*DEPDC5, NPRL2, NPRL3*), which inhibit mTOR, collectively represent the most common monogenic cause of NAFE [50]. Inheritance is autosomal dominant with a penetrance of around 60% [44].

The GATOR1-related focal epilepsies encompass a broad spectrum of lesional and non-lesional epilepsies (including the autosomal dominant familial epilepsy syndromes) (see below and Table 1). GATOR1-related focal epilepsies are frequently drug-resistant and are associated with an increased risk of sudden unexpected death in epilepsy (SUDEP) [50]. MCD are seen in around a quarter [49, 50], most commonly focal cortical dysplasia (FCD) Type II, although others are also seen [49]. Dysplasias may be subtle on conventional neuroimaging or only identified histologically following resection [44, 50]. Brain somatic variants in mTOR pathway genes are a common and important cause of FCD Type II and hemimegaloencephaly [5, 51], including in individuals with pathogenic germline mTOR variants, where they provide a “second-hit” limited to the affected tissue [51, 52].

Identification of a pathogenic GATOR1 variant can have important clinical implications: It may encourage further brain imaging to look for subtle dysplasias, raise awareness of important prognostic information such as the possible increased risk of SUDEP, suggest favourable epilepsy surgery outcomes, or highlight potential gene-driven future therapies such as mTOR pathway inhibitors [49] (see section on Gene-driven therapies for adults with monogenic epilepsies).

### Familial Focal Epilepsy Syndromes

The NAFEs include several familial syndromes for which monogenic causes have been identified (Table 1). These syndromes are genetically heterogeneous, with both monogenic and polygenic contributions. Most identified monogenic forms are autosomal dominant, but penetrance is incomplete, and family histories may not always be available [53].

Monogenic aetiologies are identified in more than 80% of individuals with self-limited neonatal and infantile focal epilepsies [55], but these are infrequently encountered in adult epilepsy clinics as seizures, by definition, remit during childhood in the majority. In some, however, seizures can occur during adolescence and adulthood [56]. Some individuals with self-limited infantile epilepsy due to pathogenic *PRRT2* variants also develop paroxysmal kinesigenic dyskinesia (PKD), which can present for the first time in adolescence or early adulthood [57]. Dyskinesias in PKD may be misdiagnosed as focal seizures or as psychogenic episodes [58], but are important to recognise as dyskinetic episodes, as they respond well to low doses of sodium channel-blocking antiseizure medications (ASMs).

The remaining familial focal epilepsy syndromes have a variable age of onset and frequently persist into adulthood even when onset is in childhood [53, 54]. Monogenic causes are found less frequently than in the self-limited neonatal and infantile epilepsies, particularly in sporadic cases (i.e. without a family history of epilepsy) (Table 1).

I. Autosomal Dominant Sleep Related Hypermotor Epilepsy (ADSHE)
  A monogenic aetiology is identified in approximately 19% of individuals with ADSHE (i.e. where there is a known family history of sleep related hypermotor epilepsy (SHE) and/or other epilepsy) but in only 7% of sporadic SHE (i.e. where there is no family history of epilepsy) [59]. Pathogenic variants in a number of genes are associated with ADSHE (Table 1), including in genes encoding nicotinic acetylcholine receptor subunits, and in the GATOR1 complex genes where focal cortical dysplasia may also be identified in some individuals [59]. Pathogenic variants in *KCNT1* are associated with a severe form of ADSHE with comorbid intellectual disability and psychiatric features [60].
II. Autosomal Dominant Epilepsy with Auditory Features (ADEAF)
  Epilepsy with auditory features (EAF) most commonly occurs sporadically but can be familial. Pathogenic variants are identified in 15-50% of families with ADEAF [61–63], with variants in LGI1 or RELN most commonly described [62, 64]. More recent studies, utilising broader genetic testing strategies, suggest that EAF may be more genetically heterogeneous, with causal variants in other epilepsy-associated genes also described, including *DEPDC5 *and* SCN1A*. A few families with ADEAF have been reported to carry variants in *MICAL1* [65]; however, a definitive gene-disease association for *MICAL1* has yet to be established[12]. Monogenic causes are rarely identified in of sporadic cases [61, 66, 67] (Table 1).
III. Familial Mesial Temporal Lobe Epilepsy (FMTLE)
  FMTLE is a common focal epilepsy syndrome with onset in adolescence or early adulthood. Brain imaging is typically normal, although a subset have hippocampal atrophy/sclerosis [68]. The genetic architecture of FMTLE is polygenic with complex inheritance in most [68, 69], and monogenic causes are rare [70] (Table 1).
IV. Familial Focal Epilepsy With Variable Foci (FFEVF)
  FFEVF is characterized by focal seizures originating from different cortical regions in different family members, but each affected person within a family experiences only one specific type of focal seizure [54]. Seizure severity can vary significantly between family members, and some may have comorbid neurodevelopmental disorder or psychiatric disease [71]. Brain imaging may be normal or may identify an (FCD) (which may be subtle) [72]. Pathogenic variants in *DEPDC5*, as well as other GATOR1 complex genes, have frequently been reported in FFEVF families (both with and without FCD identified on brain imaging) [44, 71, 72]. In some individuals with FFEVF and pathogenic GATOR1 complex variants, FCD (typically FCD Type II) may be identified histologically, despite a normal brain imaging [44, 72]. The proportion of FFEVF families where a monogenic cause is identified is unknown, largely due to small study sizes, biases in study population selection, and challenges with obtaining large enough pedigrees to fulfil diagnostic criteria for FFEVF (i.e. demonstrating a family history of individuals with focal seizures that arise from cortical regions that differ between family members). In one small study of five families with FFEVF, three (60%) were found to have a pathogenic variant in *DEPDC5* [73].

### Other Focal Epilepsies

Seemingly acquired causes of focal epilepsy may also have an underlying monogenic aetiology, such as porencephalic cysts associated with pathogenic variants in *COL4A1* and *COL4A2*[74]. Structural lesions which may result in focal epilepsy may also have a monogenic aetiology (e.g. cavernoma and arteriovenous malformations [75]).

### Progressive Myoclonic Epilepsies

The progressive myoclonic epilepsies (PMEs) are rare disorders characterised by progressive myoclonus alongside varying degrees of neurocognitive decline, ataxia, seizures, and other neurological, neuropsychiatric or systemic features, often with premature mortality. Onset is typically in late childhood or adolescence but can present for the first time in adulthood. Early in the disease course, PMEs may be difficult to distinguish from more common genetic generalised epilepsies, particularly juvenile myoclonic epilepsy. Features such as background slowing on EEG, drug-resistance, and progressive neurological decline should prompt consideration of a PME, and further investigation, including genetic testing, should be undertaken [76].

A genetic diagnosis can be established in around 80% of individuals with PME, making PMEs one of the most genetically well-defined epilepsies [77, 78]. Almost fifty genes have now been reported to be associated with the PMEs [79]. This includes genes associated with the most common “classical” PMEs (e.g. Unverricht‐Lundborg disease (*CSTB*), Lafora disease (*EPM2A, NHLRC1*), neuronal ceroid lipofuscinoses (*TPP1, DNAJC5, CLN5, CLN6*) and myoclonic epilepsy with ragged red fibres (*MT-TK*)), rarer PME genes reported in only a small number of individuals, and genes typically associated with other epilepsy phenotypes but where PME phenotypes have also been reported (e.g. *CHD2*) [77–79]. Genetic testing utilising NGS should be undertaken when a PME is suspected [80, 81]. However, it should be noted that repeat expansions (an important cause of some PMEs (e.g. Unverricht‐Lundborg disease (*CSTB*) and dentatorubral-pallidoluysian atrophy (*ATN1*)), may not be well captured by standard sequencing‐based tests, and the application of specific bioinformatic tools (e.g.ExpansionHunter) to sequencing data may be required [82]. Alternatively, testing utilising polymerase chain reaction (PCR) and Southern blot methods should be undertaken where necessary. Variants in mitochondrial DNA are also associated with PME (e.g. *MT-TK* and myoclonic epilepsy with ragged red fibres) and mitochondrial DNA sequencing (or analysis) may not be undertaken as standard unless specifically requested.

### Familial Adult Myoclonic Epilepsy (FAME)

Familial adult myoclonic epilepsy (FAME) is a rare, autosomal dominant disorder with onset in adolescence or adulthood. It is characterised by cortical myoclonus (presenting as a postural and kinetic hand tremor, often resembling essential tremor), myoclonic seizures, and infrequent generalised tonic–clonic seizures [83]. Whilst the disease course is more favourable than in the PMEs, slowly progressive worsening of myoclonus, and occasionally other neurocognitive features, can be seen [84].

FAME is caused by a noncoding intronic pentanucleotide repeat expansion within in one of six distinct genes (*SAMD12, STARD7, MARCHF6, YEATS2, TNRC6A,* and *RAPGEF2*) [85]. The repeat expansion is comprised of a TTTTA repeat, which is expanded compared to the reference sequence, and an inserted TTTCA repeat which is not observed in non-affected populations [85]. Longer repeat expansions are associated with earlier disease onset and more severe disease, and anticipation has been described in some families [85]. Genetic testing for these repeat expansion disorders is with either PCR and Southern blot techniques, or with NGS with application of the appropriate bioinformatic tools (see PME section above).

There are currently no gene-driven therapies available for management of FAME, and treatment is based on use of medications targeting seizures and cortical myoclonus.

### Developmental and Epileptic Encephalopathies

Among the epilepsies, gene discovery has had the most significant impact in DEEs and other early-onset epilepsies. The DEEs comprise a heterogenous group of rare, severe, neurodevelopmental disorders, typically beginning in infancy or childhood, and characterised by drug-resistant epilepsy, developmental delay and/or regression, and other comorbidities[2]. Developmental impairment in the DEEs is a consequence of both the underlying aetiology (e.g. genetic, structural or metabolic), and superimposed epileptic activity[2].

NGS studies in children with DEEs and other early onset epilepsies have shown that a monogenic aetiology can be identified in 24–55% [86–90]. These findings, amongst others, have helped to revolutionise the investigation, diagnosis, and management of children with epilepsy. Yet the translation of this into the care of adults with epilepsy has been slow, despite up to 74% of children with early-onset monogenic epilepsies estimated to go on to require neurological care into adulthood [21]. NGS sequencing studies of adults with epilepsy and neurodevelopmental disorders (including DDEs) show genetic diagnostic yields that are comparable to paediatric cohorts, ranging from 23–50%, and have provided valuable information about the monogenic epilepsies that may be encountered (and diagnosed) in selected adults with epilepsy [91–99] (Supplementary Table 1). For many adults with epilepsy and intellectual disability, details of the early clinical history may be lacking, and characteristic features typical in certain epilepsy syndromes may be less apparent during adulthood, making syndromic diagnoses (and the potential therapeutic opportunities that brings) challenging [24]. The majority of adults alive today with a DEE, other early-onset epilepsy, or neurodevelopmental disorder with epilepsy will not have benefited from recent genetic diagnostic discoveries, and, in adults with compatible phenotypes, genetic testing should be considered (see below) [22–24]. Many DEEs remain epileptic encephalopathies in adulthood, with persistent seizure burdens likely contributing to poor cognitive outcomes [24]. This further highlights the importance of exploring genetic aetiologies, which may uncover potential gene-driven therapeutic strategies.

Across studies of adults and children with epilepsy undergoing genetic testing, *SCN1A* is the most ubiquitous epilepsy-associated gene [94] (Supplementary Table 2). *SCN1A* loss-of-function variants cause Dravet syndrome (the most common monogenic DEE), whereas rarer gain-of -function variants lead to an early infantile DEE with hyperkinetic movement disorder and other features [100]. Pathogenic variants in *SCN1A* are also associated with genetic epilepsy with febrile seizures plus (GEFS +) [101] and some NAFEs [61]. Across 14 adult NGS studies (Supplementary Table 1 and 2), variants in *SCN1A* accounted for 15% of all genetic diagnoses made, and were identified in 2% of all adults who underwent genetic testing, a relatively high proportion for a single causal gene. This is an important finding, as it highlights that despite *SCN1A*-related epilepsies (such as Dravet syndrome GEFS +) being some of the most widely recognised syndromes, many individuals may remain undiagnosed into adulthood, and adult neurologists should still suspect these, and other monogenic epilepsies, as potential diagnoses to be made in adults with compatible phenotypes [22, 23]. It is important to note that, whilst a causal variant is not required for the diagnosis of Dravet syndrome and many other epilepsy syndromes, in adults where details of the early clinical history are lacking, or where characteristic features may be less apparent, a genetic finding can help in supporting—or suggesting—the clinical diagnosis [23].

### Monogenic Epilepsies Identified in NGS Studies in Adults

To further evaluate which monogenic epilepsies may be encountered in adult epilepsy clinics, a literature search in PubMed was undertaken (compiled June 2023), using the search terms in Box 1. The authors identified 17 published diagnostic NGS studies of adults with epilepsy, encompassing 3887 individuals (Supplementary Table 1 and 2). A summary of these studies is presented alongside results from selected NGS studies in children with epilepsy (Supplementary Table 2). Whilst these studies are not directly comparable due to differences in both the studied populations and the genetic testing methodologies employed, the outcomes are presented together here to illustrate findings across cohorts and to highlight potential patterns of commonality and divergence.

Across adult NGS studies, the diagnostic yield ranged from < 3% to 50%. This wide variation may be largely explained by heterogeneity in the population characteristics, with studies including adults with early onset epilepsies and neurodevelopmental disorders (e.g. DEEs) showing higher diagnostic yields compared to cohorts mainly comprised of individuals with NAFE. Use of different genetic testing methodologies across studies may also contribute to differences in diagnostic yield, with yields highest for WGS, followed by WES, followed by gene panel testing [102].

**Box 1** Search terms used for literature review on diagnostic NGS in adults with epilepsy (Compiled June 2023):

**Table:** 

“Adult”
AND
“Epilepsy” or “Seizure” or “Epileptic”
AND
“Genetic” or “Gene panel” or “Genome” or “Exome”

The references in identified papers and any relevant citing articles were also reviewed as part of the literature search

**Fig. 1:**
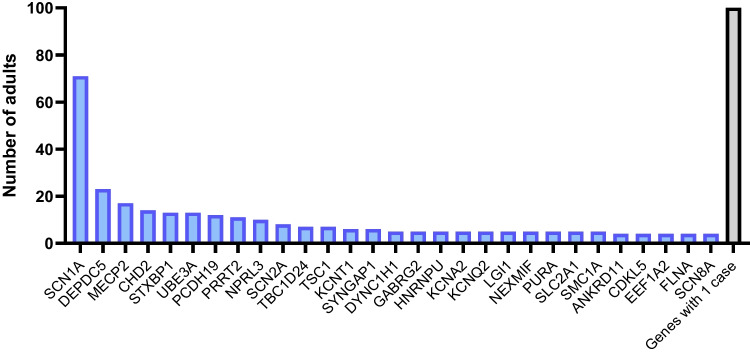
The distribution of causal genes identified in adults with epilepsy from 14 published NGS studies. Only genes in which ≥ 4 cases were reported in the literate are shown. For genes in which < 4 cases were reported in the literature see Supplementary Fig. 1. Genes in which only one adult case has been described in NGS studies are shown (*N* = 100)

The most commonly implicated causal genes (across 14 studies which reported individual gene data) include *SCN1A, DEPDC5, MECP2, CHD2, STXBP1,* and *UBE3A*, together accounting for around a third of all genetic diagnoses made (Supplementary Table 2; Fig. 1). These findings reveal a clear overlap in the causal genes that account for the most common monogenic epilepsies identified through testing in both childhood and adulthood across selected populations (Supplementary Table 2). They also highlight notable differences. Variants in *KCNQ2* are one of the most common monogenic causes of epilepsy in children [86, 103], but are not encountered frequently in adults undergoing genetic testing (Supplementary Table 2) [95]. *KCNQ2*-associated epilepsies encompass a wide spectrum of epilepsy syndromes, including self-limited early-onset epilepsies (where seizure remission is seen in the majority of affected children [56]), and *KCNQ2*-DEE (where seizure freedom is not uncommon by adolescence or early adulthood [104]). Children with early-onset self-limiting epilepsies, or those who have been seizure-free for many years, are less likely to transition into adult epilepsy clinics, meaning that these syndromes may not be encountered as frequently by adult neurologists [95]. Those cases that *are* encountered in adult clinics are likely to be those at the more severe end of the disease spectrum and represent an important cohort of individuals for understanding the full spectrum, natural history and prognosis of these monogenic epilepsies.

Genes associated with monogenic NAFE featured more commonly in adult NGS studies compared to most paediatric/mixed-age cohorts (Supplementary Table 2). *DEPDC5* and *NPRL3* were two of the most common monogenic causes of epilepsy across 14 NGS studies in adults with epilepsy, together accounting for 7% of all genetic diagnoses made (Supplementary Table 2, Fig. 1). There are several methodological factors that may contribute to these differences. Some paediatric/mixed-age studies pre-date the discovery of the GATOR1 complex genes (roles in epilepsy described for *DEPDC5* in 2013 [105] and *NPRL2/3* in 2016 [43]), and paediatric/mixed-age studies do not included *DEPDC5* or *NPRL3* in the gene panels tested, including the largest study (involving 8565 individuals [106]) included in the 24 combined NGS studies reported by Symonds et al. [103]. Conversely, most adult NGS studies have been conducted since 2020, and include the GATOR1 complex genes within the tested gene panels. In addition, several adult NGS studies have focused on NAFEs where GATOR1 complex genes may be expected to be identified more commonly than in other epilepsy cohorts (see below). It is also important to note that in the GATOR1-related epilepsies, the age of seizure onset can range from early infancy through to late adulthood [49], and as such, some individuals with these (and other) monogenic focal epilepsies, may present for the first time to adult epilepsy clinics.

Early diagnosis and improvements in care mean that most children with severe early-onset epilepsy syndromes now survive into adulthood [21]. However, there is an increased risk of death amongst children with epilepsy compared to a matched population without epilepsy [107], and children with severe syndromes, such as the DEEs, may be disproportionately affected [108]. For example, it is estimated that 10–20% of children with Dravet syndrome die before reaching adulthood, mostly due to SUDEP [109]. As such, some monogenic epilepsies may be seen less frequently in adult epilepsy clinics due to increased rates of childhood mortality. Natural history and long-term follow-up studies extending into adulthood are lacking for most monogenic epilepsies, and there is a paucity of literature about features of the monogenic epilepsies in adulthood, even for the most well-characterised syndromes [110]. As a result, information about life expectancy, mortality, and outcomes in adulthood for the monogenic epilepsy syndromes is largely unknown [21]. Pathogenic variation in the gene *CDKL5* was the third most common cause of monogenic epilepsy identified across 24 NGS studies mainly involving children [103] (Supplementary Table 2). The largest study included in this combined analysis of 24 studies involved 8565 individuals. Causal *CDKL5* variants were identified in 99/8565 individuals, representing 7.5% of the total genetic diagnoses made [106]. Of note, although the cohort included adults and children, the median age at the time of genetic testing of the individuals with causal *CDKL5* variants was 1.8 years [106]. Similarly, in a population-based study of children with epilepsy onset before 36 months, *CDKL5* was one of the most common monogenic causes of epilepsy, accounting for 5% of all genetic diagnoses made [86]. Conversely, across 14 NGS studies in adults, causal *CDKL5* variants were identified in only 4 individuals, representing < 1% of all genetic diagnoses made. *CDKL5* deficiency disorder is a DEE characterised by infantile-onset drug-resistant seizures, severe developmental delay, cortical visual impairment and other comorbidities [111]. Very few adults with *CDKL5* deficiency disorder are reported in the literature, and little is known about long-term outcomes, prognosis, and mortality. SUDEP is reported in *CDKL5* deficiency disorder, but the risk and frequency of SUDEP in this monogenic epilepsy is unknown [111]. Whilst we acknowledge that this is only an observation, and that there may be multiple factors that account for the differences in the proportion of individuals identified with *CDKL5* deficiency disorder in childhood compared to adulthood, it illustrates the need for greater understanding of the adult phenotype, natural history, long-term outcomes, prognosis, and mortality in the early-onset monogenic epilepsies.

## Which Adults Should be Offered Genetic Testing?

In adults with epilepsy and intellectual disability/neurodevelopmental disorder, in particular where epilepsy onset is in early childhood, genetic diagnostic yields are comparable to paediatric cohorts [91–99], reinforcing the point that the use of genetic diagnostic testing should not be limited by age in adults with these phenotypes. Lower diagnostic yields are seen in those with NAFE, but vary according to the epilepsy syndrome, and presence/absence of a family history (Table 1, Supplementary Table 1). Whilst diagnostic yields may be lower, the absence of a family history of epilepsy should not *absolutely* dissuade from pursuing a genetic aetiology in individuals with NAFE [41], as de novo variation and reduced penetrance are both observed. Genetic testing is not currently recommended for the routine diagnostic evaluation of IGEs, as the aetiology is rarely monogenic. In some cases, however, genetic testing may be considered, particularly where there are additional unexplained features such as intellectual disability, developmental delay, dysmorphism, or movement disorder, a progressive course and/or unexplained cognitive decline (see section on PME), drug-resistance, or a family history suggesting dominant inheritance [25, 33, 80]. Although still rare, glucose transporter 1 deficiency disorder (associated with variants in *SLC2A1*) is the most common monogenic cause of IGE, and making a diagnosis may have important management implications, including initiating treatment with the ketogenic diet [33].

Based on diagnostic yield, as well as clinical utility, genetic testing has been recommended for individuals with epilepsy who have one of the conditions described in Box 2 [80, 81].

**Box 2** Genetic testing is recommended (or should be considered) in adults with epilepsy who fall into the following groups:

**Table:** 

• Developmental and epileptic encephalopathy (or other severe childhood-onset epilepsies)
• Comorbid neurodevelopmental disorder (e.g. intellectual disability or autism spectrum disorder)
• Other comorbidities that may suggest a genetic aetiology (e.g. dysmorphism, congenital malformations)
• The presence of a malformation of cortical development
• Those with a familial focal epilepsy syndrome
• NAFE with either a positive family history, comorbid neurodevelopmental disorder, or drug-resistance (particularly if undergoing presurgical evaluation[112])
• Progressive myoclonic epilepsies or other progressive syndromes
• Features suggestive of a mitochondrial or metabolic disorder

Recommendations adapted from Krey et al. [80] and Pickrell et al. [81]

A range of genetic testing modalities are available, and the choice of test will depend on the clinical scenario, as well as the potential options available for use in the local healthcare setting. Each test has advantages and disadvantages, with differing diagnostic yield when utilised in people with epilepsy [102]. A recommended genetic testing strategy for an individual with epilepsy, in whom a genetic aetiology is suspected, is to utilise WES or WGS as a first-line test, or a comprehensive multigene panel if WES or WGS are not available. If these tests are non-diagnostic, CNV analysis should be considered [113]. For some individuals, targeted genetic testing may be required in addition to, or in place of, the above strategies, such as triplet repeat expansion testing for Fragile X syndrome, or karyotyping when rearrangements or ring chromosomes are suspected [80, 113].

Currently, genetic testing in clinical practice is predominantly used for diagnostic purposes. However, the increasing utilisation of WGS across healthcare settings provides an opportunity to harness genomic data not only for diagnostics, but also to inform individualised treatment choice based on pharmacogenomic factors [114], or for predicting risk of comorbid conditions [115]. By leveraging genomic data to its fullest potential, WGS may be utilised more widely to inform management of epilepsy, offering the possibility of individualised gene-driven management [114].

## Gene-Driven Approaches to Treatment

One of the many benefits that a genetic diagnosis can bring to an individual with epilepsy is that it opens up the possibility of treatments that are guided by knowledge of the specific underlying molecular genetic aetiology and disease pathophysiology. The promise of this treatment approach, which has been referred to as “precision medicine”, “personalised medicine”, or “gene-driven therapy”, is that it allows for more individualised treatments, either by targeting the precise underlying cause of the disease, or by selecting (or avoiding) treatments that have evidence for their effectiveness (or lack of efficacy) in a specific disease or syndrome. The aim of gene-driven therapies for the monogenic epilepsies is to improve seizure control, but also to bring about improvements in (or prevention of) other aspects of the disease, including cognitive, developmental, psychiatric, and other neurological or non-neurological comorbidities, ultimately resulting in improved quality of life and improved survival (e.g. through a reduction in the risk of SUDEP) [80]. Gene-driven therapies may fall into one of five categories which are detailed in Box 3.

**Box 3** Types of gene-driven therapies:

**Table:** 

1. Substitutive/replacement therapies: These long-established therapies are largely used in the management of inherited metabolic epilepsies and involve the substitution or replacement of deficient components of the involved biochemical pathways. For example, the use of pyridoxine in the management of pyridoxine-dependent epilepsy secondary to variation in the gene *ALDH7A1* [116]
2. Evidence-based use of ASMs (non-targeted or targeted): A genetic or syndromic diagnosis may aid in the selection (or avoidance) of ASMs that have been shown to be more (or less effective/contraindicated) for the treatment of seizures in people with a given syndrome. Evidence for the use of a particular ASM may come from randomised controlled trials (RCTs), N-of-1 trials, case series, and/or other studies, and may include established ASMs or novel/repurposed drugs with proven anti-seizure effects (e.g. fenfluramine [117, 118]). In some cases, the ASM may be targeted to the specific underlying disease mechanisms (e.g. the use of sodium channel-blocking ASMs for the treatment of seizures in individuals with *SCN2A*-related epilepsies due to gain-of-function variants [119]). In other instances, the ASM may not directly target the underlying disease pathophysiology, but it may have proven efficacy in individuals with a particular epilepsy syndrome (e.g. cannabidiol for the treatment of seizures in Dravet Syndrome [120–122]). In some individuals, a genetic diagnosis may also help to inform decision-making surrounding epilepsy surgery [123–126]
3. Novel or repurposed targeted therapies: Targeted therapies are those that are directed towards the specific underlying pathophysiological mechanisms of a given monogenic epilepsy. They may include repurposed or novel therapies that have not been primarily developed as ASMs. These therapies do not act through genetic manipulation, or modulation of gene expression (see genetic therapies below), but act to reverse the specific functional impacts of the pathogenic variant at the protein level, with a view to preventing or improving the symptoms associated with that functional defect. Evidence for the use of a novel or repurposed targeted therapy may come from RCTs, N-of-1 trials, case series, and/or other studies. An example is the use of 4-aminopyridine for the treatment of *KCNA2*-related DEE secondary to variants that result in gain-of-function of the encoded potassium channel [127]
4. Disease-modifying gene therapies: These novel therapies are currently in various stages of development for several of the monogenic epilepsies. Although there are no disease-modifying gene therapies available for use in monogenic epilepsies currently, several are already undergoing clinical trials (e.g. Phase 1/2 open-label trial of an antisense oligonucleotide (STK-001) [128], and a Phase 1/2 trial of an adeno-associated virus-9 based gene therapy (ETX101) [129], both in children with *SCN1A*-related Dravet syndrome. Gene therapies aim to target and correct a well-defined pathogenetic mechanism through the introduction of new genetic material, genome modification, or modulation of gene expression, with the intention of alleviating (or ideally preventing) all aspects of the disease phenotype
5. Therapies directed by pharmacogenomics: In all individuals with epilepsy, regardless of the underlying aetiology, genetic factors can contribute to response to ASMs (and potentially gene-driven therapies more broadly), both in terms of efficacy and adverse effects [130]. Whilst not routinely employed currently, utilisation of genomic data should extend beyond diagnostics to inform individualised choice of ASM based on pharmacogenomic factors, for example, testing for risk-conferring HLA variants to mitigate the risk of ASM-induced cutaneous adverse drug reactions [114]

Of course, gene-driven management extends far beyond selection of the most appropriate medications or surgical strategies. A genetic diagnosis can bring an end to the diagnostic odyssey [131] (which for adults may be many decades long), providing families with answers, alleviating parental guilt, and improving family quality of life [132]. Connection with syndrome or gene-specific advocacy groups is also facilitated, which can offer much needed support, information, and resources for families. A diagnosis may enable more informed knowledge of the future evolution and progression of an individual’s epilepsy, as well as the potential associated comorbidities that may arise. It enables the appropriate investigations and screening to be undertaken and helps families and clinicians to be aware of, and prepare for, potential challenges that may arise. Furthermore, a diagnosis may also have implications for reproductive choices, and the need for genetic counselling both for the individual with epilepsy and the wider family. Importantly, genetic diagnoses made in adulthood contribute invaluable information which widens our understanding of the disease phenotype and course of these rare conditions beyond childhood and offer important insights into disease outcomes in the absence of long-term follow-up and natural history studies.

It is important to recognise that undergoing genetic testing and receiving a genetic diagnosis can have a considerable psychosocial impact on individuals with epilepsy, their parents and caregivers, and it is essential that individuals have access diagnosis-specific information, and genetic counselling when required [133].

## Gene-Driven Therapies for Adults with Monogenic Epilepsies

Across 17 published NGS studies in adults with epilepsy, a genetic diagnosis reportedly had implications for clinical management in 10–71% of individuals (Supplementary Table 1). These included selection/avoidance of specific ASMs, initiation of metabolic therapies, exploration of possible surgical interventions, and awareness of the need for additional investigations and screening for associated comorbidities [42, 93, 95–97, 134]. The wide variation between studies in the proportion of individuals for whom a genetic diagnosis reportedly had management implications is likely due to differing standards of evidence accepted for various gene-driven treatment approaches. For example, in a study by Zacher et al., a gene-driven treatment approach was identified in 40% of individuals with monogenic epilepsy. However, a high level of evidence (i.e. randomised controlled trial (RCT) level supporting the use of a particular gene-driven therapy was available for only a quarter of these cases—equating to just 10% of those with a monogenic epilepsy overall [96]. The majority of studies did not report the outcomes of any gene-driven management strategies that were instituted (Supplementary Table 1), so their efficacy and tolerability in this adult population could not be evaluated. Only two studies reported treatment outcomes [42, 95], one of which reported that in 17% of those in whom a genetic diagnosis was made, gene-driven treatment changes led to an improvement in seizure control and/or cognitive function [95] (Supplementary Table 1). There is mounting evidence from anecdotal reports and small case series that a genetic diagnosis made in adulthood can be beneficial, and that it is “never too late” to pursue a genetic aetiology in an adult with epilepsy [93]. Reports in the literature where positive outcomes were seen following treatment changes instigated as a result of a genetic diagnosis made in adulthood include those for pathogenic variants in *SCN1A *[22, 42, 95, 135]*, SLC2A1 *[95]*, GAMT *[136]*, ALDH7A1 *[137]*, KCNA2 *[127]*, ARG1 *[135]*,* and *DEPDC5 *[135], and there are likely many others (both published and unpublished). Positive outcomes described after gene-driven treatment changes made in adulthood include: improvements in seizure frequency, cognition, language/communication, quality of life, and other aspects of the disease, and may be seen into the 7th decade of life, even after many decades of drug-resistant seizures and treatment with contraindicated medications [22].

Many review articles have provided comprehensive summaries of the ever-expanding list of existing and emerging gene-driven therapies for the monogenic epilepsies [123, 138, 139]. In Table 2 we highlight the gene-driven therapies that are available or currently undergoing clinical trials for the ten most common monogenic epilepsies identified in adults with epilepsy, which account for more than a third of all genetic diagnoses made across 14 published NGS studies (Supplementary Table 2). It is important to note that some of the therapies listed below relate to treatment of clinically defined syndromes (such as Dravet and Rett syndrome) regardless of the underlying genetic aetiology (e.g. Lorcaserin for Dravet syndrome), whereas other therapies are both syndrome and gene-specific therapies (e.g. STK-001 for *SCN1A*-related Dravet syndrome).

**Table 2:**
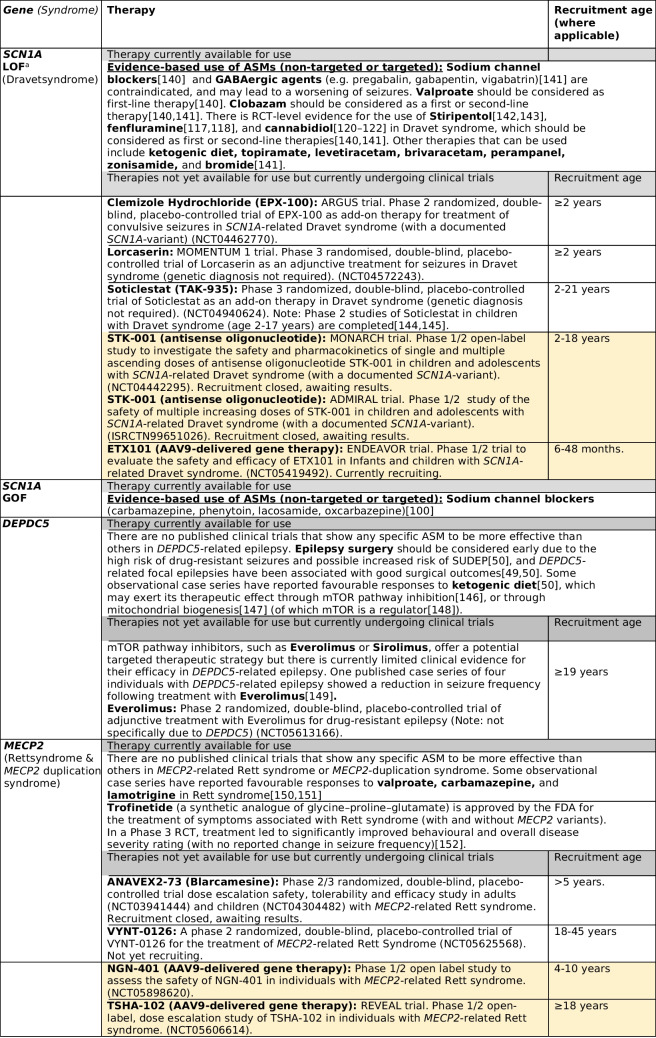

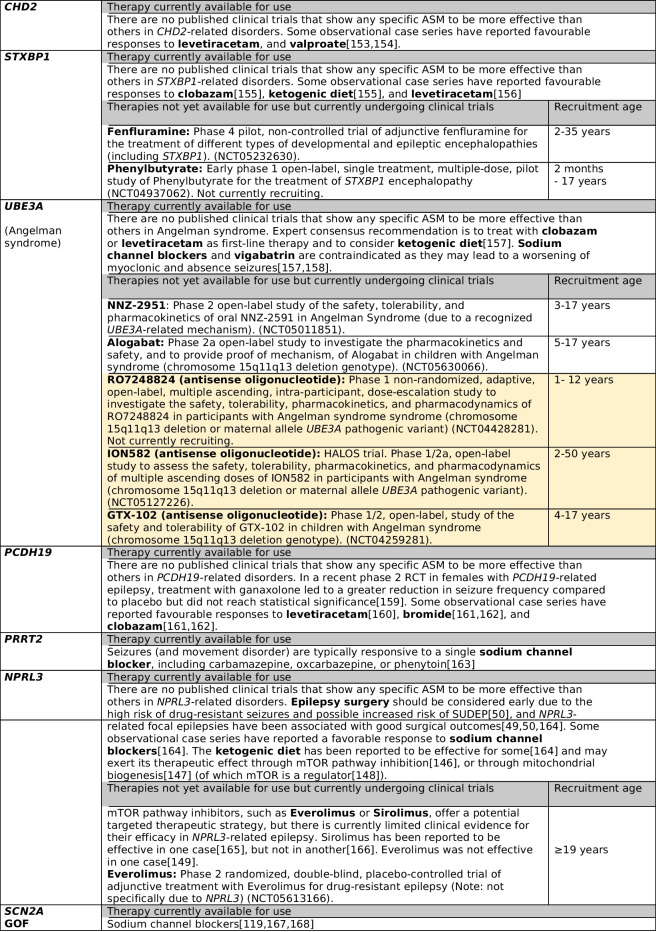
Gene-driven therapies for the ten most common monogenic epilepsies identified across adult NGS studies

Included clinical trials are those that are currently recruiting, about to recruit, or which have recently closed recruitment but results are awaited (correct at the time of writing (August 2023). Therapies from completed and published trials which are not currently licensed for clinical use are not included here*.* ^a^Evidence for these therapies comes from studies of individuals with Dravet syndrome, and there is limited evidence for the use of these therapies in other *SCN1A*-related epilepsies due to loss of function variants. Of note, these therapies are also recommended for individuals with Dravet syndrome who do not have known *SCN1A* variants (i.e. in those with other monogenic causes of Dravet syndrome, or in those in whom a genetic cause has not been identified); AAV9 = adeno-associated viral vector serotype 9; ASM = antiseizure medication; GOF = gain of function; LOF = loss of function; NGS = next generation sequencing; RCT = randomised controlled trial; SUDEP = sudden unexpected death in epilepsy. Yellow highlight = disease-modifying gene therapies

For the majority of monogenic epilepsies there are currently no available targeted therapies, and disease-modifying gene therapies are still in development or undergoing clinical trials. Evidence for gene-driven therapies that *are* currently available comes largely from open label trials, anecdotal reports, retrospective case series, and in vitro functional studies, with very few gene-driven therapies supported by evidence from RCTs or N-of-1 trials [80, 123, 138]. In addition, the long-term outcomes following the use of gene-driven therapies (in comparison to “standard therapy”) are yet to be documented [80]. Whilst there has been much anticipation and hope from the promise of gene-driven therapies in monogenic epilepsies, the real-world experience is complex, littered with unexplained treatment failures, and complicated by heterogeneous treatment responses [135, 169] (even amongst individuals with the same underlying causal variant [127]). The reasons for this have not been fully ascertained – but this is essential if we are to consider treatments ‘precision’. An additional challenge in the management of adults with monogenic epilepsies is that most published reports of the efficacy and tolerability of gene-driven therapies come from studies in children. As such, the efficacy, side effect profile, risks, and potential outcomes are largely unknown in the adult population. For example, Dravet syndrome is one of the few monogenic epilepsies for which there is evidence from RCTs for the efficacy of specific ASMs (Table 2). However, all published RCTs in Dravet syndrome to date, including for stiripentol [142, 143], cannabidiol [120–122], and fenfluramine [117, 118], only included children (age 2–18 years). Published data on the use of these medications specifically in adults with Dravet syndrome is lacking and is limited to small observational case series (stiripentol [170, 171], cannabidiol [172], fenfluramine [173]). From these observational studies, there is some evidence to suggest that response rates to stiripentol [170] and cannabidiol [172] may be less favourable when initiated in adulthood compared to their use during childhood. In addition, adverse events may be seen more commonly when cannabidiol [174] and fenfluramine[173] are initiated in adulthood compared to childhood. Several clinical trials of novel therapies for use in people with Dravet syndrome are now recruiting adults (Table 2). It is important to note, however, that trials of disease-modifying gene therapies in Dravet syndrome, including of the antisense oligonucleotide STK-001 and adeno-associated viral vector serotype 9-delivered gene therapy ETX101, have only included children.

Similar concerns have been highlighted regarding the efficacy and tolerability of gene-driven therapies for the treatment of adults with other monogenic epilepsies. This includes the use of quinidine for the treatment of *KCNT1*-gain-of-function (GOF) epilepsies where an age-dependent therapeutic response has been suggested, with older age at treatment associated with lack of efficacy [175], and 4-aminopyridine for the treatment of *KCNA2*-GOF DEE, where younger age at initiation is associated with a more favourable treatment response [127].

These concerns highlight some of the challenges of using gene-driven therapies in adults with monogenic epilepsies, and whilst they should not discourage their use, it is important that clinicians be cautious about raising expectations about treatment outcomes [135], particularly where robust evidence about their efficacy in adults is lacking. It is also clear that adults should be included in trials of gene-driven therapies, not least for equity.

## The Challenges of Gene-Driven Therapies in Adults with Monogenic Epilepsies

There are numerous benefits to identifying a genetic diagnosis in an individual with epilepsy, and whilst there is much anticipation and hope from the promise of gene-driven therapies in the monogenic epilepsies, there remain significant challenges in clinical implementation [135, 169], some of which have already been discussed. Whilst many of these challenges are evident for both children and adults with monogenic epilepsies, some may be magnified in adults.

### Greater Knowledge of Monogenic Epilepsies in Adults is Needed

Many adults currently being managed in epilepsy clinics today will have missed out on opportunities for genetic testing during childhood, particularly with NGS which has only become available in clinical practice in many countries in recent years, and access to genetic testing may remain limited in adult neurology and epilepsy clinics, particularly in resource-poor settings. There remains a paucity of published literature on the monogenic epilepsies in adulthood, even for the most well-described syndromes [110], and data on treatment response, including to gene-driven therapies, are sparse. This may be partly due to insufficient use of genetic investigations in adults with epilepsy, with many now calling for more widespread uptake of genetic testing in this population [23, 93, 134]. In order to evaluate the efficacy and tolerability of gene-driven therapies in adults with monogenic epilepsies, we need to understand more about the adult phenotype, include adults in clinical trials and other studies of novel therapies, and make available information about successful and unsuccessful responses to gene-driven therapies. Syndrome or gene-specific registries offer a useful opportunity to gather clinical data, including treatment-response, over time, and are being employed or developed for a number of monogenic epilepsies [176].

### Gene Therapies – when to Treat. Is Adulthood too Late?

Gene therapies for a number of monogenic epilepsies are currently being assessed in preclinical human cell and animal models, with some already undergoing clinical trials (see Table 2 for examples). An important consideration for the use of gene therapies for monogenic epilepsies is whether there is a “therapeutic window” during which ‘normalisation’ of the target gene can rescue and/or prevent the associated disease [177]. Many genes that are associated with monogenic epilepsies play an essential role in nervous system development [177], and variants in some genes may activate epileptogenic mechanisms through their impact on neurodevelopment [123]. Genes harbouring pathogenic variants (and their encoded proteins) do not exist or function in insolation. A pathogenic variant in one gene, or abnormal function of the encoded protein, may bring about secondary (including homeostatic) changes in the expression of other genes, which in turn may also contribute to the disease phenotype [178]. As such, a gene therapy that targets only an individual implicated gene may not have sufficiently wide-reaching effects to reverse all aspects of the resulting complex underlying pathophysiology, particularly if the gene therapy is delivered later during disease development (such as in adulthood) when potentially pervasive changes in gene-expression, neuronal development, and dysfunctional neural networks are already established. Thus, for some diseases, it is possible that gene therapies may need to be administered during embryonic development in order to rescue all aspects of the disease phenotype [123, 138, 177, 178]. However, given that variants in many epilepsy-associated genes show incomplete penetrance, even for those associated with severe DEEs [179], this approach would not currently be justifiable and the principle needs discussion in society more widely. The “therapeutic window” of a gene therapy is likely to be different for each gene and may depend on a number of factors including the temporal pattern of gene expression, whether the gene has a direct (or indirect) role in early neurodevelopment, and whether it continues to have an essential role into adult life.

There is some evidence from animal models of monogenic epilepsies that delivery of gene therapies (or other means of normalisation of target gene expression) after symptom onset can rescue the disease phenotype, including in mouse models of *SCN1A*-related Dravet syndrome [180], *UBE3A*-associated Angelman syndrome [181], and *MECP2*-associated Rett syndrome [182]. These models lend support to the idea that, at least for some genes, there may be arguments for administration of gene therapies after symptom onset and into adult life [183]. This may not result in a full reversal of the disease phenotype but may still have the potential to bring about clinical benefits that are meaningful for the person with epilepsy. In the DEEs, seizures and interictal discharges contribute to developmental, cognitive, and behavioural impairments, in addition to the impact of the causal variant. In these devasting, early-onset disorders, seizures are typically refractory to standard ASMs, meaning that ongoing seizures throughout life may continue to have detrimental effects on long-term outcomes. Assessment of adults with DEEs show that many still have ‘active’ epileptic encephalopathies into adulthood [24]. Whilst delivery of a gene therapy during adulthood may not be able to reverse the full developmental phenotype, improved seizure control in an adult with an active epileptic encephalopathy may result in clinically meaningful benefits. However, formal studies including adults are needed.

### “Monogenic” isn’t Really Monogenic

There is growing evidence that the genetic architecture of the “monogenic” epilepsies (and other “monogenic” neurodevelopmental disorders) may be more complex than initially suspected, with variation beyond the causal coding or genic variant contributing to the disease phenotype [4, 185, 186]. Individuals with established (or presumed) monogenic DEEs, have an excess of rare variants in known epilepsy-associated genes [4, 187], and other genes [187, 188], as well as an increased polygenic risk for epilepsy (conferred by common genetic variation) [186]. Significant differences in common genetic variation have also been identified across disease-relevant traits, such as intelligence and longevity, in individuals with *SCN1A*-related Dravet syndrome [4]. Multiple molecular genetic diagnoses (i.e. a pathogenic variant at more than one genetic locus, each associated with an independent disease), are identified in between 3.2–7.2% of individuals undergoing genetic testing [189], and causal variants in more than one epilepsy-associated gene have been described in several individuals, with contributions from each implicated gene resulting in a “blended phenotype” [4, 41, 96, 189]. The contribution of this “background” genetic variation likely partly explains phenomena such as genetic pleiotropy (a spectrum of syndromes or severity of disease reportedly associated with one gene), and reduced penetrance (variable expression of the disease phenotype in individuals harbouring the same variant), which are observed in many monogenic epilepsies [185, 186, 190, 191]. As the complexities of the “monogenic” epilepsies unravel, it is becoming apparent that digenic, oligogenic, polygenic, dual molecular diagnoses, and double-hit contributions to disease pathogenesis, and the resulting phenotype, need to be considered [4, 169, 186, 192]. This complex, multifaceted genetic architecture to the “monogenic” disorders will raise questions about how “targeted” or “precise” gene-driven therapies should (or could) be, and whether it is plausible to expect that the full disease phenotype can truly be reversed by targeting only one gene (because disease is not in the end caused by one gene).

## Conclusions

Genetic factors play an important contribution to the aetiology of epilepsy and should be considered early in the presentation. Many adults with epilepsy will not have benefited from genetic testing in childhood, particularly with NGS technologies which have only recently become available for clinical use. In some individuals with epilepsy, variation within a single gene (monogenic) has a major contribution to the overall clinical phenotype. Identifying a monogenic cause for an individual’s epilepsy may have important implications for management, including in the potential for gene-driven therapies. The yield of genetic diagnostic testing in adults with epilepsy and neurodevelopmental disorders is similar to that in children, ranging from 23–50%. Outside of the familial focal epilepsy syndromes diagnostic yields in the NAFE are low (< 12%). However, genetic testing should still be considered, particularly in those with an associated MCD, family history, or drug-resistance, and in those undergoing pre-surgical evaluation.

An ever-expanding list of gene-driven therapies is emerging, although very few are supported by evidence from formal studies. Most studies of gene-driven therapies have included only children, and several ongoing trials of disease-modifying gene therapies in monogenic epilepsies have limited recruitment to those under 18 years. Thus, for many gene-driven therapies, the efficacy and tolerability in adults is largely unknown. However, anecdotal reports and small case series have reported beneficial effects of gene-driven therapies in adults with monogenic epilepsies, even after many decades of drug-resistant seizures. This emphasises the importance of undertaking genetic testing in adults with epilepsy, as well as widening participation in clinical trials and other studies to include adults.

The genetic architecture of the “monogenic” epilepsies is emerging as increasingly complex, with potential genome-wide contributions to the disease pathophysiology and resulting phenotype. This adds further complexity to the development of gene-driven therapies, in particular disease modifying gene therapies, and questions whether it is plausible to expect that the full disease phenotype can truly be reversed by targeting only a single gene.

## Supplementary Information

Below is the link to the electronic supplementary material.

Supplementary file1 (DOCX 594 KB)
