# Supplementary material for: Monogenic Epilepsies in Adult Epilepsy Clinics and Gene-Driven Approaches to Treatment
Source: Curr Neurol Neurosci Rep. 2025 May 17;25(1):35. doi: 10.1007/s11910-025-01413-x (PMC12085364; doi:10.1007/s11910-025-01413-x)
Supplement: Supplementary file 1 — Supplementary file1 (DOCX 594 KB) [file 11910_2025_1413_MOESM1_ESM.docx]

**Supplementary material**

**Supplementary Figure 1: The distribution of causal genes identified in adults with epilepsy from 14 published NGS studies (including genes in which <4 cases were reported)**


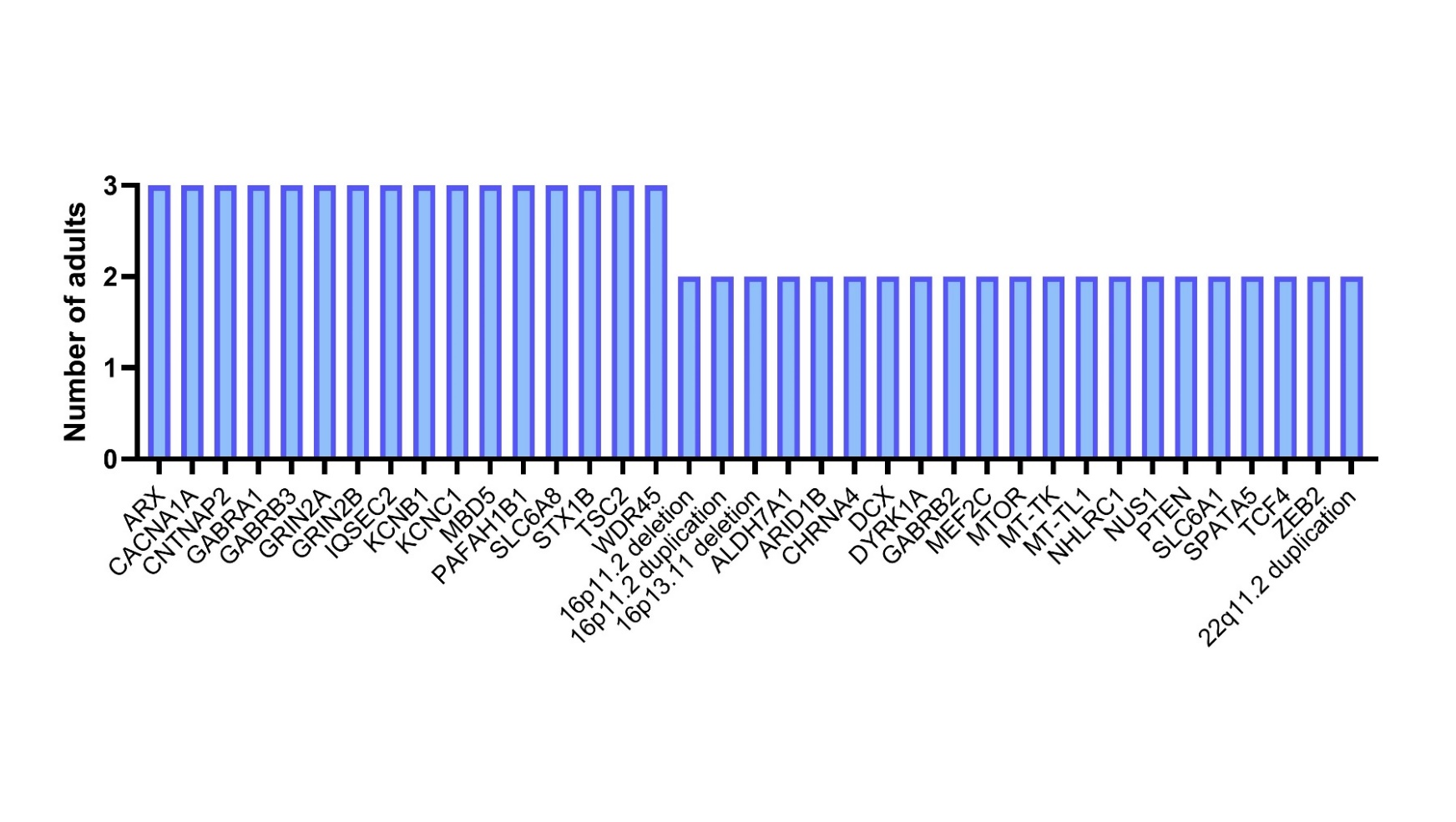


**Supplementary Figure 1: The distribution of causal genes and copy number variants identified in adults with epilepsy from 14 published NGS studies.** Genes in which <4 cases were reported in the literate are shown. For genes in which ≥4 cases were reported in the literature see Figure 1 in main text.

**Supplementary Table 1: Diagnostic NGS in adults with epilepsy**

| Reference  Total number of adult cases  Country/year of data collection | Cohort  Age range of cohort at time of genetic testing (years) | Panel / testing | Variant classification criteria used | Diagnostic yield | Gene-driven therapy available (as reported by study authors) |
| --- | --- | --- | --- | --- | --- |
|  |  |  |  |  | Outcome from gene-driven therapy (as reported by study authors) |
| McKnight 2022^1a^  N=2008  USA/2015-20 | Adults with epilepsy referred for genetic testing^b^  Age: 18 – 90 | Epilepsy panel (89–189 genes) with SNV and CNV analysis^c^ | Sherloc a proprietary, points-based framework based on ACMG | 218/2008 (11%) | 121/218 (55.5%) |
|  |  |  |  |  | Outcomes not provided |
| Leu 2020^2^  N=448  USA | Adults with focal (N=393) or generalised (N=55) epilepsy. Comorbidities included depression anxiety and other mental health conditions, no NDD/ID. 20% - 36% had a family history of epilepsy  Age: range not provided. Mean 39.7 (focal epilepsy), 38.2 (generalised epilepsy) | Genome-wide CNV and whole-exome SNV analyses. Reviewed genes associated with “neurological or psychiatric disease”) | ACMG | 13/448 (<3%) | Not reported by authors |
| Krey 2022^3e^  N=232  Germany | Adults with epilepsy and ID  Age: not provided | WES with SNV and CNV analysis | ACMG | 107/232  (46%) | Not reported by authors |
| Johannesen 2020^4^  N=200  Denmark/2013-18 | Adults with epilepsy “referred for genetic diagnostics”  91% had ID.  Age: 18 – 80 | Epilepsy/ID/ASD panel (45-580 genes)^c^ | ACMG | 46/200 (23%) | Total number not reported by authors |
|  |  |  |  |  | 8/46 (17%) treatment change led to clinical benefit |
| Li 2022^5^  N=164  Canada/2016-21 | Adults with epilepsy of unknown aetiology (89%) or associated with malformations of cortical development or other structural lesions  10% had ID, 80% drug-resistant epilepsy, 86% had focal epilepsy, 11% had a family history of epilepsy  Age: not provided | Epilepsy panel (36 – 144 genes)^d^ and/or mitochondrial nuclear gene panel (141 genes), PME panel (18 genes), IGE panel (91 genes) | In-house criteria with pathogenicity “determined by the team responsible for the genetic testing” | 7/164  (4%) | 5/7 (71%) |
|  |  |  |  |  | Outcomes not provided |
| Zacher 2021^6^  N=150  Germany/2017-19 | Adults with neurodevelopmental disorders with epilepsy  Age: 18 – 84 | Karyotyping, *FMR1* testing, microarray, gene panel (4811 genes^c^), +/- single or trio WES | ACMG | 71/150 (47%) | 7/71 (10%)^g^  high evidence^h^ in epilepsy-associated genes  23/71 (30%) moderate evidence^i^ in epilepsy-associated genes |
|  |  |  |  |  | Outcomes not provided |
| Guo 2021^7^  N=121  USA/2015-20 | Adults with a diagnosis of epilepsy without a known genetic cause, referred to the neurogenetics clinic^b^  Age: 16 – 87 (5 cases <18 years) | Targeted candidate gene, microarray, mitochondrial genome sequencing (from whole blood), gene panel^e^, WES | A variant was deemed causative if classified as “pathogenic” or “likely pathogenic” by the clinical laboratory. | 36/121  (30%) | A few anecdotal reports provided only. |
|  |  |  |  |  | Outcomes not provided |
| Jiang 2020^8^  N=118  China/2015-19 | Adults with “genetically unexplained epilepsy” sequentially recruited from a tertiary epilepsy centre  17% had ID/DD, 21.5% had a family history of epilepsy, median age at seizure onset was 12 years, 24.5% focal epilepsy, generalised epilepsy 50%  Age: 18 – 50 | Gene panel (153 genes)^c^ or WES | ACMG | 12/118 (10%) | Not reported by authors |
| Krenn 2020^9^  N=112  Austria | Adults with non-lesional focal epilepsy (excluding hippocampal sclerosis), AND at least one of: family history of seizures; seizure onset <25 years; ASM resistance. Exclusion: moderate-severe ID  51% temporal lobe epilepsy; 33% had a family history, 90% non-lesional, 10% hippocampal sclerosis/atrophy; 77% drug-resistant  Age: ≥18 | WES including exome-based CNV analysis (virtual epilepsy panel of 455 genes)^c^ | ACMG and Baldassari criteria for GATOR-complex genes | 13/112 (12%) | A few anecdotal reports provided only. |
|  |  |  |  |  | Outcomes not provided |
| Benson 2020^10^  N=70    Ireland | Adults with unexplained refractory epilepsy and ID  Age: 18 – 55 | Trio-WES (virtual panel 166 epilepsy-associated genes)^c^ and microarray (61/74) | ACMG | 19/70  (27%) | 3/19 (16%) |
|  |  |  |  |  | Outcomes not provided |
| Minardi 2020^11^  N=71  Italy/2016-17 | Adults with DEE of unknown aetiology  100% childhood onset seizures, 35% dysmorphic features, 15.5% brain malformations  Age: 21 – 65 | WES (virtual epilepsy/ID panel of 2375 genes)^j^ | ACMG | 18/71 (25%) | 9/18 (50%) |
|  |  |  |  |  | Outcomes not provided |
| Snoeijen-Schouwenaars 2019^12f^  N=53  Netherlands | Adults with epilepsy and ID of unknown cause  Epilepsy onset in neonatal, infancy or childhood in 79%  Age: 18 – 67 | WES (with virtual epilepsy (269 genes)^c^ +/- ID panel (835 genes))  If the panel analysis did not reveal a pathogenic variant, the diagnostic exome sequencing was extended to the full exome | ACMG | 15/53 (28%) | Not reported separately for adult cases |
| von Brauchitsch 2023^13^  N=52 | Adults with epilepsy and ID of unknown cause  Median age of seizure onset 3.0 years (range 0-26), 65% drug resistant, 33% family history of epilepsy  Age: 20 – 57 | WES with virtual panel of 2364 epilepsy and developmental genes^c^ | ACMG | 16/52 (31%) | Not reported by authors |
| Bardakjian 2018^14f^  N=38  USA | Adults with epilepsy referred for genetic diagnostic evaluation^b^  Age: 19 – 69 | Targeted candidate gene, gene panel^e^, microarray, WES | Criteria not stated | 6/38  (16%) | Not reported by authors |
| Perucca 2017^15^  N=28  Australia/2014 | Non-lesional focal epilepsy and family history of epilepsy  Age: 18 – 74 | WES (virtual epilepsy panel of 64 genes)^c^ | ACMG | 3/28  (11%) | 1/3 (33%) |
|  |  |  |  |  | 1/1 (100%) treatment change led to clinical benefit |
| Lemke 2012^16^  N=14  Germany and Switzerland | Adults with a range of epilepsy syndromes but without a genetic diagnosis. Included syndromes where monogenic yield would be expected to be high (e.g. Dravet syndrome (5 cases))  Age: 22 – 53 | Epilepsy panel (265 genes)^c^ | In-house criteria (note pre-ACMG) | 6/14  (43%) | Not reported by authors |
| Thevenon 2016^17^  France  N=8 | Adults with an epileptic encephalopathy or severe to profound non-syndromic ID and epilepsy  Age:18 – 34 | WES | In-house criteria (note pre-ACMG) | 4/8  (50%) | Not reported separately for adult cases |

^a^3.2% of the cohort included in McKnight 2022 were included previously in Borlot *et al*.^18^ and 52% were included in a study focused on childhood-onset epilepsy^19^; ^b^Further detailed clinical information not provided; ^c^panel includes *CDKL5;* ^d^four out of the five epilepsy panels used included *CDKL5*;^e^information about genes included in panel not provided; ^f^individual gene data not provided for this cohort (not included in Figure 1, Supplementary Figure 1, Supplementary Table 2); ^g^excludes two cases in which only secondary genetic findings (not related to the epilepsy phenotype) were identified; ^h^when reporting authors applied the highest “centre for evidence based medicine” (CEBM) levels (I and II) to a potential gene-driven therapy approaches; ^i^when reporting authors applied moderate CEBM level (III) to potential gene-driven therapy approaches; ^j^90% of individuals had already undergone prior genetic tests (karyotype, microarray, single gene or gene-panel testing), with negative findings; Green highlight = studies focusing on individuals with focal epilepsies. ACMG=American college of medical genetics; ASD=autism spectrum disorder; DEE=developmental and epileptic encephalopathy; ID=intellectual disability; IGE=idiopathic generalised epilepsy; NDD=neurodevelopmental disorder; NGS = next generation sequencing; PME=progressive myoclonic epilepsy; WES=whole exome sequencing; WGS=whole genome sequencing

**Supplementary Table 2: Results from selected NGS studies in children and adults with epilepsy**

|  | **Population-based cohort study**  **Symonds 2019**^20^ | **Summary of 24 NGS studies**  **Symonds 2020**^21^ | **Largest single NGS study**  **Truty 2019**^19^ **^a^** | **Largest single NGS study including only children**  **Yang 2019**^22^ **^a^** | **Summary of 14^b^ NGS studies including only adults ^c^** |
| --- | --- | --- | --- | --- | --- |
| **Number of individuals included** | 333 | 13,063 | 9769 | 733 | 3564 |
| **Age of the cohort included** | Children only  Age: <36 months | Mixed-age  Includes 24 studies with a range of ages (mostly children) | Mixed-age  “Most individuals <5 years of age”. Median age: 6 years (range: 0‐82). 10.7% were adults at the time of testing^1^ | Children only  Ages not given | Adults only  ≥18 years^c^ |
| **Phenotypic description of included cases** | Population-based study. Children <36months with a diagnosis of epilepsy, prolonged febrile seizure, or febrile status | Includes 24 studies with a range of inclusion criteria | Epilepsy | Children with severe seizures, with age of onset <1 year. Excluded those with known CNVs | Includes 14 studies of adults with epilepsy with a range of inclusion criteria (See **Supplementary Table 1** for details) |
| **Genetic testing method** | 104-gene panel | Includes 24 studies using a range of NGS (+/- other modalities) | 183-gene panel | 2742-gene panel or WES | Includes 14 studies using various NGS (+/- other modalities) |
| **Diagnostic yield** | 24% | 17% | 15% | 32% | 16% |
| **Ten most commonly implicated genes in each cohort**  *(Percentage of total positive genetic diagnoses given for genes discussed further in the main text)* | *PRRT2*  *SCN1A (18%)*  *KCNQ2 (13%)*  *SLC2A1*  *CDKL5 (5%)*  *PCDH19*  *SLC6A1*  *DEPDC5 (5%)*  *KCNQ3*  *KCNA2* | *SCN1A (>19%)*  *KCNQ2 (>10%)*  *CDKL5 (>7%)*  *SCN2A*  *STXBP1*  *PCDH19*  *PRRT2*  *SCN8A*  *MECP2*  *SLC2A1* | *SCN1A (16%)*  *MECP2*  *PRRT2*  *KCNQ2 (7%)*  *DEPDC5 (4%)*  *PCDH19*  *SCN2A*  *STXBP1*  *UBEA3*  *SYNGAP1* | *SCN1A (17%)*  *TSC2*  *KCNQ2 (9%)*  *STXBP1*  *SCN2A*  *CDKL5 (3%)*  *PCDH19*  *PRRT2*  *MECP2*  *DEPDC5 (1.5%)* | *SCN1A (15%)*  *DEPDC5 (5%)*  *MECP2*  *CHD2*  *STXBP1*  *UBE3A*  *PCDH19*  *PRRT2*  *NPRL3 (2%)*  *SCN2A* |
|  |  |  |  |  | *(KCNQ2 (1%))*  *(CDKL5 (<1%))* |

^a^ Studies not included in summary of 24 NGS studies by Symonds *et al.* ^21^; ^b^Three studies (323 individuals) are excluded (Bardakjian *et al*.^14^, Snoeijen-Schouwenaars *et al*.^12^ Krey *et al*.^3^) as individual gene data is not provided within paper; ^c^Includes five individuals age 16-17. Yellow highlighted column = includes 14 NGS sequencing studies in adults with epilepsy, identified by the authors following a literature review using the search terms in **Box 1,** and combined by the authors to evaluate the spectrum of monogenic epilepsies across an adult epilepsy population. Full details of each included adult study can be found in **Supplementary Table 1**. NGS = next generation sequencing and refers to targeted gene panel sequencing, whole exome or whole genome sequencing (typically with the application of a virtual panel).

**References**

1. McKnight, D. *et al.* Multigene panel testing in a large cohort of adults with epilepsy. *Neurol. Genet.* **8**, e650 (2022).

2. Leu, C. *et al.* Neurological disorder-associated genetic variants in individuals with psychogenic nonepileptic seizures. *Sci. Rep.* **10**, 15205 (2020).

3. Krey, I., Johannesen, K. M., Kohnen, O. & Lemke, J. R. Genetic testing in adults with developmental and epileptic encephalopathy – what do we know? *Med. Genet.* **34**, 207–213 (2022).

4. Johannesen, K. M. *et al.* Utility of genetic testing for therapeutic decision-making in adults with epilepsy. *Epilepsia* **61**, 1234–1239 (2020).

5. Li, J. *et al.* Usage of genetic panels in an adult epilepsy clinic. *Can. J. Neurol. Sci.* 1–7 (2022).

6. Zacher, P. *et al.* The genetic landscape of intellectual disability and epilepsy in adults and the elderly: a systematic genetic work-up of 150 individuals. *Genet. Med.* **23**, 1492–1497 (2021).

7. Guo, M. H. *et al.* Temporal trends and yield of clinical diagnostic genetic testing in adult neurology. *Am. J. Med. Genet. A* **185**, 2922–2928 (2021).

8. Jiang, Y.-L. *et al.* Clinical utility of exome sequencing and reinterpreting genetic test results in children and adults with epilepsy. *Front. Genet.* **11**, 591434 (2020).

9. Krenn, M. *et al.* Diagnostic exome sequencing in non-acquired focal epilepsies highlights a major role of GATOR1 complex genes. *J. Med. Genet.* **57**, 624–633 (2020).

10. Benson, K. A. *et al.* A comparison of genomic diagnostics in adults and children with epilepsy and comorbid intellectual disability. *Eur. J. Hum. Genet.* **28**, 1066–1077 (2020).

11. Minardi, R. *et al.* Whole-exome sequencing in adult patients with developmental and epileptic encephalopathy: It is never too late. *Clin. Genet.* **98**, 477–485 (2020).

12. Snoeijen-Schouwenaars, F. M. *et al.* Diagnostic exome sequencing in 100 consecutive patients with both epilepsy and intellectual disability. *Epilepsia* **60**, 155–164 (2019).

13. von Brauchitsch, S. *et al.* The phenotypic and genotypic spectrum of epilepsy and intellectual disability in adults: Implications for genetic testing. *Epilepsia Open* **8**, 497–508 (2023).

14. Bardakjian, T. M. *et al.* Genetic test utilization and diagnostic yield in adult patients with neurological disorders. *Neurogenetics* **19**, 105–110 (2018).

15. Perucca, P. *et al.* Real-world utility of whole exome sequencing with targeted gene analysis for focal epilepsy. *Epilepsy Res.* **131**, 1–8 (2017).

16. Lemke, J. R. *et al.* Targeted next generation sequencing as a diagnostic tool in epileptic disorders. *Epilepsia* **53**, 1387–1398 (2012).

17. Thevenon, J. *et al.* Diagnostic odyssey in severe neurodevelopmental disorders: toward clinical whole-exome sequencing as a first-line diagnostic test. *Clin. Genet.* **89**, 700–707 (2016).

18. Borlot, F. *et al.* Clinical utility of multigene panel testing in adults with epilepsy and intellectual disability. *Epilepsia* **60**, 1661–1669 (2019).

19. Truty, R. *et al.* Possible precision medicine implications from genetic testing using combined detection of sequence and intragenic copy number variants in a large cohort with childhood epilepsy. *Epilepsia Open* vol. 4 397–408 Preprint at https://doi.org/10.1002/epi4.12348 (2019).

20. Symonds, J. D. *et al.* Incidence and phenotypes of childhood-onset genetic epilepsies: a prospective population-based national cohort. *Brain* **142**, 2303–2318 (2019).

21. Symonds, J. D. & McTague, A. Epilepsy and developmental disorders: Next generation sequencing in the clinic. *Eur. J. Paediatr. Neurol.* **24**, 15–23 (2020).

22. Yang, L. *et al.* Clinical and genetic spectrum of a large cohort of children with epilepsy in China. *Genet. Med.* **21**, 564–571 (2019).
